# Insulin Resistance and Muscle Metabolism in Chronic Kidney Disease

**DOI:** 10.1155/2013/329606

**Published:** 2013-02-03

**Authors:** James L. Bailey

**Affiliations:** Renal Division, Emory University School of Medicine, Woodruff Memorial Research Building, Room 338, 1639 Pierce Drive, Atlanta, GA 30322, USA

## Abstract

Insulin resistance is a common finding in chronic kidney disease (CKD) and is manifested by mild fasting hyperglycemia and abnormal glucose tolerance testing. Circulating levels of glucocorticoids are high. In muscle, changes in the insulin signaling pathway occur. An increase in the regulatory p85 subunit of Class I phosphatidylinositol 3-Kinase enzyme leads to decreased activation of the downstream effector protein kinase B (Akt). Mechanisms promoting muscle proteolysis and atrophy are unleashed. The link of Akt to the ubiquitin proteasome pathway, a major degradation pathway in muscle, is discussed. Another factor associated with insulin resistance in CKD is angiotensin II (Ang II) which appears to induce its intracellular effects through inflammatory cytokines or reactive oxygen species. Skeletal muscle ATP is depleted and the ability of AMP-activated protein kinase (AMPK) to replenish energy stores is blocked. How this can be reversed is discussed. Interleukin-6 (IL-6) levels are elevated in CKD and impair insulin signaling at the level of IRS-1. With exercise, IL-6 levels are reduced; glucose uptake and utilization are increased. For patients with CKD, exercise may improve insulin signaling and build up muscle. Treatment strategies for preventing muscle atrophy are discussed.

## 1. Introduction

Insulin resistance describes a physiological condition which is characterized by reduced tissue responses to the action of insulin for any given blood concentration of the hormone. It is a common finding in chronic kidney disease but it largely goes unrecognized. In nondiabetic patients with end stage renal disease, this is manifested by mild fasting hyperglycemia and abnormal glucose tolerance testing during an oral or intravenous glucose load. Patients may develop hyperglycemia or maintain normoglycemia at the expense of hyperinsulinemia [1, 2]. These changes are often masked by a decline in the metabolic clearance of insulin that occurs as the glomerular filtration rate drops below 15 to 20 mL/minute. Between glomerular filtration rates of 20 to 40 mL/minute, peritubular insulin uptake increases to maintain renal insulin clearance [3]. In uremia, degradation of insulin in nonrenal tissues such as liver and muscle is impaired and the half-life of insulin is prolonged. It is hypothesized that accumulation of uremic toxins may inhibit insulin degradation particularly by the liver. Although the latter is responsible for removal of approximately 50% of the insulin secreted into the portal circulation [4], the major site of insulin resistance is in the peripheral tissues. Because adipose tissue is responsible for disposal of less than 2% of the glucose load, muscle tissue is the primary site for insulin resistance. DeFronzo et al. [1] demonstrated that leg glucose exchange as a measure of peripheral insulin-mediated glucose uptake was decreased in patients with end stage renal disease and showed that a decrease in leg glucose exchange correlated with a decrease in total body insulin-mediated glucose uptake. 

## 2. The (PI3K)/Akt Pathway

**Figure 1:**
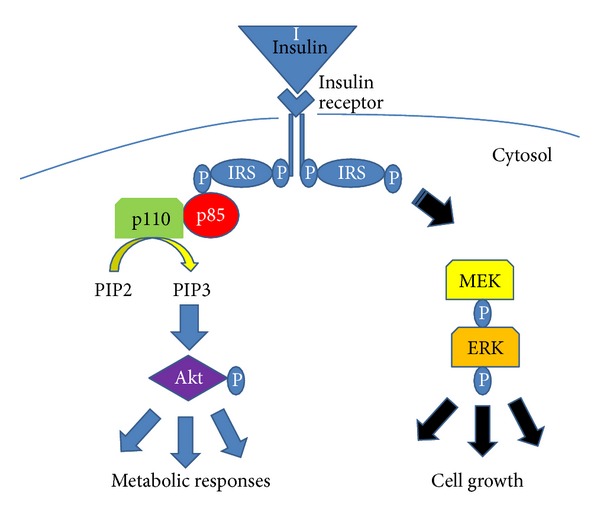
Insulin or insulin-like growth factor 1 (IGF-1) binds to its receptor and activates the receptor tyrosine kinase. The receptor undergoes autophosphorylation and provides a binding site for the insulin receptor substrate (IRS) proteins. Once bound, these IRS proteins undergo phosphorylation on tyrosine residues. These phosphorylated tyrosine residues provide a docking site for the p85 regulatory subunit of the Class I phosphatidylinositol 3-kinase. In turn, the p110 catalytic subunit is released, becomes activated and catalyzes the production of phosphatidylinositol (3, 4, 5)-triphosphate (PIP_3_) from phosphatidylinositol (3, 4)-biphosphate (PIP_2_). PIP_3 _then activates protein kinase B (Akt). Akt then serves as a branch point for a variety of downstream signaling pathways. Insulin and IGF-1 can also stimulate cell growth through the mitogen activated protein kinase pathway/extracellular signal related kinase (MEK/ERK) pathway.

 The major pathway by which insulin mediates its metabolic effects is through the Class I phosphatidylinositol 3-Kinase (PI3K)/protein kinase B (Akt) pathway. In muscle (see Figure 1), the pathway begins by insulin (or insulin growth factor 1 (IGF-1)) binding to their respective receptors (IR) and activating an internal receptor tyrosine kinase. As a result, the receptor undergoes autophosphorylation and provides a binding site for the insulin receptor substrate (IRS) proteins. In muscle, there are two major isoforms of the IRS proteins, IRS-1 and IRS-2. Each of these IRS proteins can independently bind to the phosphorylated tyrosine residues on the IR. Once bound, these IRS proteins become substrates for the IR kinase and undergo phosphorylation on tyrosine residues. In turn, these phosphorylated tyrosine residues in the IRS proteins provide a docking site for the Class I PI3K which is composed of a p85 regulatory and p110 catalytic subunits. The docking of the p85 regulatory subunit with the phosphorylated tyrosine residues of the IRS proteins results in an active enzyme complex. Subsequently, the PI3K catalyzes the production of phosphatidylinositol (3, 4, 5)-triphosphate or PIP_3_ which in turn directly activates the serine kinase Akt or indirectly via 3-phosphoinositide-dependent protein kinase (PDK). Akt serves as a branch point for a variety of downstream signaling pathways.

 The basis for the impaired responses to insulin has been obscure until fairly recently.

Cecchin and colleagues examined insulin binding and the kinase activity of insulin receptors in isolated skeletal muscles from control and uremic rats [5]. They found no change in the receptor number, insulin affinity, or tyrosine kinase activity. Similarly, Tsao and colleagues measured IGF-1 receptor number, binding affinity, and kinase activity in isolated muscles from control and chronic kidney disease (CKD) rats and reported no differences in receptor number, insulin affinity, or tyrosine kinase activity [6]. The latter finding is important because both the insulin and IGF-1 receptors transduce into common signaling pathways. Consequently, both groups concluded that the inability of insulin and IGF-1 to ameliorate muscle wasting in CKD was due to postreceptor defects. In contrast, Ding et al. reported that CKD was associated with an increase in the number of IGF-1 receptors in muscle; however, the IGF-1 receptor kinase activity was lower [7]. All of these groups used in vitro measurements which may not accurately reflect the physiological characteristics of the receptors in vivo. 

 Bailey and colleagues tested muscles of CKD and respective control rats in the insulin receptor substrate (IRS)/PI3K/Akt pathway under basal physiologic conditions and after maximum stimulation of the signaling pathway through an injection of a supraphysiologic dose of insulin [8]. The IRS proteins, PI3K and the more distal effectors of insulin/IGF-1 action, Akt were evaluated. They found functional abnormalities in the IRS/PI3K cascade that reduced the activation of the downstream effectors Akt. Specifically, the basal activity level of the IRS-1-associated PI3K as well as the phosphorylated Akt was low. Interestingly, these abnormalities were overcome when the signaling pathway was maximally stimulated with insulin. These in vivo studies are important because reductions in these signaling events have been shown to stimulate protein degradation in muscle [9–11].

 Although the p85 regulatory subunit forms a stable high affinity complex with the p110 catalytic component, the amounts of these subunits in rat muscle are differentially regulated by stimuli such as glucocorticoids [12]. Giorgino et al. reported that the expression of the p85 protein could be markedly increased in L6 muscle cells by the addition of dexamethasone [13] while P110 protein content was only modestly raised. As a result, IRS-1-associated PI3K activity was reduced. This caused the authors to propose that competition between the free p85 subunit and the PI3K enzyme complex at the binding site on IRS-1 caused the decrease in PI3K activity. IRS-1-associated PI3K activity in rat muscle has been shown to be reduced by glucocorticoids [12, 14]. This is important because glucocorticoids have been shown to be increased in the setting of CKD with or without metabolic acidosis [15]. Based on these studies, Bailey and colleagues concluded that glucocorticoids could contribute to the defects in PI3K activity in CKD rat muscle by increasing the amount of the PI3K p85 subunit but not the p110 protein [8].

## 3. Akt Isoforms

 The suppression of IRS-1 PI3-kinase activity has a variety of ramifications on cellular functions because the downstream effector of PI3-kinase activity is Akt. Akt is a serine/threonine kinase and is activated by a variety of growth factors by both PI3-Kinase dependent [16] and independent mechanisms [17, 18]. Akt appears to play a critical role in regulating skeletal muscle growth and metabolism. There are three isoforms: Akt-1/PKB*α*, Akt-2/PKB*β*, and akt-3/PKB*γ*; each isoform is encoded by a distinct gene [19]. Akt-1 is expressed ubiquitously. With Akt-1 deficiency, body size is small. There is marked impairment in growth, but glucose homeostasis is normal [20]. Akt-2 is expressed in insulin-responsive tissues such as liver, adipose tissue, and skeletal muscle. The absence of Akt-2 results in diabetes with impaired glucose tolerance and reduced insulin-dependent glucose uptake in adipose tissue and skeletal muscle while hepatic glucose production is increased [21]. Akt-3 is primarily expressed in brain and testes [22], and its deletion results in a reduction in brain size [22]. These data suggest that Akt-2 is most important in the regulation of metabolism while Akt-1 controls growth, but overlapping functions among the various Akt isoforms have been reported [23]. Constitutively active Akt in mouse skeletal muscle promotes hypertrophy and prevents atrophy [24]. Akt is known to suppress apoptosis [25]. Hence, the defective IRS/PI3 K signaling found in CKD reduces the level of the PI3 K-generated product, phosphatidylinositol 3, 4, 5-triphosphate (PIP_3_), with a subsequent decrease in Akt activation.

## 4. PTEN

PIP_3_ can undergo dephosphorylation to form inactive phosphatidylinositol 4, 5-biphosphate through the activity of the phosphatase and tensin homolog deleted from chromosome 10 (PTEN). A rise in this enzyme's activity has the same effect on insulin/IGF-1 signaling as a decrease in PI3 K activity [26]. For example, a high fat diet induces insulin resistance, and PTEN activity has been shown to be increased [27] while mice with specific muscle deletion of PTEN demonstrate improved glucose homeostasis [28]. Hu and colleagues [29] studied how changes in PTEN expression participate in the regulation of muscle proteolysis pathways. The authors found that PTEN expression was decreased in acutely diabetic mice whereas it was increased in mice with chronic diabetes. A decrease in PTEN yields an increase in PIP_3_ and an increase in Akt activity. The authors offered several explanations for these findings. They noted that in normal subjects, short-term fasting could suppress PI3K/Akt signaling as proteolytic pathways were stimulated to provide gluconeogenic amino acids. In addition, PTEN is decreased in fasting to counteract the increase in muscle protein breakdown. 

In chronic diabetes or insulin resistant states, gluconeogenesis is enhanced from substrates derived from protein breakdown in muscle. A decrease in IRS-1-associated PI3K activity as well as an increase in PTEN would act in concert to lower PIP_3_. Subsequently, muscle protein breakdown would be accelerated. This process of muscle breakdown will be further discussed in subsequent sections. 

## 5. Akt Regulation of FOXO1/3

**Figure 2:**
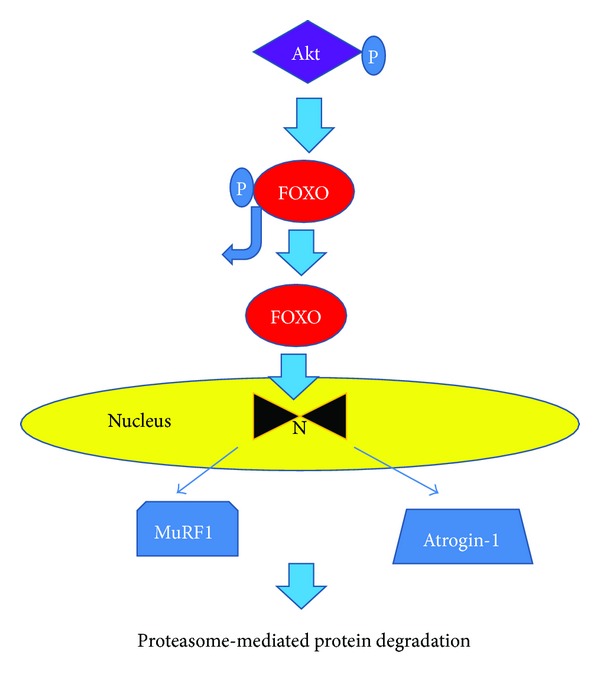
A downstream target of protein Kinase B (Akt) is the Forkhead box O or FOXO transcription factors. Phosphorylation of the FOXOs by Akt deactivates them and prevents them from translocating to the nucleus. Dephosphorylated FOXOs translocate to the nucleus where they increase the expression of a variety of genes that suppress skeletal muscle hypertrophy and result in muscle atrophy. They also induce ubiquitin ligases such as muscle ring finger-1 (MuRF1) and atrogin-1 that promote skeletal muscle proteolysis.

 One of the downstream targets of Akt is the Forkhead box O or FOXO family of transcription factors include FOXO1 and FOXO3 among others [30]. They are important regulators of metabolic processes. The FOXOs influence the transcription of genes involved in metabolism [31, 32], the cell cycle [33], and apoptosis [34]. The transcriptional activities of FOXO proteins are governed by posttranslational modifications such as phosphorylation and acetylation. Phosphorylation of the FOXOs by Akt deactivates them by preventing them from translocating to the nucleus and increasing the expression of a variety of genes that may suppress skeletal muscle hypertrophy [35]. FOXO proteins also induce ubiquitin ligases and promote proteolysis in skeletal muscle [36, 37] (see Figure 2).

## 6. Skeletal Muscle Types, FOXO and PGC1-*α*

 Although skeletal muscle appears uniform histologically, it consists of myofibers that are heterogeneous with respect to size, metabolism, and contractile function. These myofibers are classified into different types based on their expression of specific myosin heavy chains. These include type I, type IIa, type IId/x, and type IIb fibers. Type I or slow-twitch fibers are associated with type I myosin, contract slowly, are rich in mitochondria and tend to be resistant to fatigue. In contrast, type II or fast-twitch fibers, contract quickly, depend primarily on glycolysis and rapidly fatigue. Exercise training induces fiber-type changes from type IIb to type IId/x to type II*α* and type I as the myofibers are transformed and oxidative metabolism is increased. There is a dramatic increase in mitochondrial content as the result of the expression of genes that increase mitochondrial biogenesis. Muscle oxidative capacity and metabolic efficiency are enhanced. The transcriptional coactivator peroxisome-proliferator-activated receptor gamma coactivator-1*α* (PGC1-*α*) enhances mitochondrial biogenesis and oxidative metabolism by playing a key role in regulating mitochondrial gene expression. This is interesting because Kamei and colleagues [38] established transgenic mice over expressing human FOXO1. The skeletal muscle of these FOXO1 mice weighed less and gene expression for type I (red muscle) fiber was reduced. Histological examination of the skeletal muscle of these mice demonstrated fewer type I fibers and smaller type I and type II fibers, leading the authors to conclude that FOXO1 is a negative regulator of skeletal muscle mass and type 1 muscle fiber-related genes. As the FOXO1 protein can interact with PGC-1*α*, the authors speculated that FOXO1 might inhibit PGC-1*α* function by binding to it. Lin and colleagues [39] had previously shown that PGC-1*α* is expressed preferentially in muscle enriched in type 1 fibers. When PGC-1*α* was expressed at physiological levels in transgenic mice, a fiber type conversion was seen. Putative type II muscles from PGC-1*α* transgenic mice expressed proteins characteristic of type I fibers and demonstrated a much greater resistance to fatigue. PGC-1*α* and PGC-1*β* isoforms had been thought to play a role in muscle fiber type determination as well as insulin resistance. However, Zechner and colleagues studied mice with total PGC-1 deficiency in skeletal muscle [40]. These mice had a dramatic reduction in exercise capacity as characterized by diminution in muscle oxidative capacity with rapid depletion of muscle glycogen stores. They also noted that there were derangements in mitochondrial structure; however, these authors found that the proportions of oxidative muscle fiber types (I, II*α*) were not reduced nor were there alterations in insulin sensitivity and glucose tolerance. The differences noted may be related to the age or functional status of the mice when studied. Nevertheless, PGC-1*α* plays a key role in modulating the mitochondrial network and one factor that regulates muscle fiber type determination [40]. There is also significant evidence to suggest a link between skeletal muscle mitochondrial dysfunction and the development of insulin resistance [41–43]. This is particularly important in regards to the accelerated protein degradation associated with catabolic states such as chronic kidney disease and poorly controlled diabetes.

Other signaling pathways that regulate the shift in skeletal muscle fiber type have been identified and include Ras-ERK1/2 [44], calcineurin [45], and Ca^2+^/calmodulin-dependent protein kinase IV [46]. It is beyond the scope of this review to talk about the Ras-ERK1/2 pathway. Calcineurin, a calcium-dependent protein phosphatase, has two relevant substrates that it dephosphorylates, the nuclear factor of activated T cells (NFAT) and myocytes enhancer factor 2 (MEF2). NFAT and MEF2 work in concert to increase the transcription of prototypical type I oxidative muscle fiber genes that include PGC-1*α* [47]. Dephosphorylation of NFAT proteins enables their translocation from the cytoplasm to the nucleus where they bind to myocytes enhancer factors and increase their transcriptional activity [48]. There are four distinct genes that have been identified that encode closely related NFAT proteins (NFAT1-NFAT4).

They are thought to play distinctive roles in fiber type determination. NFAT1 is putatively involved in the upregulation of MyHC I gene expression during the fast to slow fiber type shift in rodent skeletal muscle cells [44]. In rats made diabetic with streptozotocin, PGC-1*α* protein and mRNA are decreased in skeletal muscle [49]. This correlates with a suppression of calcineurin activity. In addition, MEF2 and NFAT activity are substantially reduced as seen by the decrease in mRNA for their downstream target genes, myogenic regulatory factor 4 (MRF4), and modulatory calcineurin interacting protein 1.4 (MCIP1.4). Moreover, levels of MRF4, MCIP1.4, and PGC-1*α* are also decreased in muscles from mice lacking calcineurin CnA*α* ^−/−^ and CnA*β* ^−/−^. These findings suggest that decreased calcineurin signaling rather than changes in other calcium- or cAMP-sensitive pathways are responsible for decreased PGC-1*α* expression in skeletal muscle during diabetes [49]. It is probable that other conditions such as CKD, which are associated with loss of muscle mass, employ a similar mechanism. 

## 7. Glucocorticoids, Metabolic Acidosis, and **** Muscle Cachexia

 Cachexia and loss of muscle mass is a common occurrence in patients with CKD, but muscle atrophy or loss does not develop because of a lack of intake of sufficient protein and calories. Instead, protein catabolism results from a series of maladaptive responses to a series of complications which are seen with advanced disease, namely, metabolic acidosis, insulin resistance, elevated levels of angiotensin II, increased production of glucocorticoids, and inflammation [50–53]. May and colleagues found that insulin stimulation of protein synthesis was suppressed while protein degradation was increased in skeletal muscle from rats with CKD [54]. This was attributed in part to a glucocorticoid dependant mechanism [55]. The other factor was metabolic acidosis. Urinary corticosterone levels increased in proportion to the degree of metabolic acidosis and only the acidotic rats had increased rates of protein degradation. When the acidosis was corrected with the addition of sufficient alkali in the form of sodium bicarbonate, rates of protein degradation were no different from those of in control rats. Interestingly, urinary corticosterone levels remained elevated. This is important because Mak [56] showed that correction of metabolic acidosis in CKD rats only partially corrects insulin resistance. In patients with CKD not yet on dialysis, insulin resistance improves with the addition of sodium bicarbonate to correct the metabolic acidosis [57]. Moreover, insulin sensitivity was studied in 8 patients on chronic hemodialysis before and after two weeks of oral sodium bicarbonate therapy to correct the metabolic acidosis [58]. Using the hyperinsulinemic euglycemic clamp technique, insulin sensitivity and secretion increased following sodium bicarbonate therapy. Sodium chloride had no effect, suggesting that it was the addition of the alkali and not the sodium that was crucial in ameliorating the insulin resistance. Garibotto and colleagues [59] found an interesting inverse correlation between serum cortisol and bicarbonate levels in CKD patients. Lower serum bicarbonate levels were associated with higher serum cortisol levels. Moreover, higher rates of protein degradation correlated directly with serum cortisol levels and inversely with serum bicarbonate levels. 

## 8. The Ubiquitin-Proteasome System and **** Muscle Atrophy

**Figure 3:**
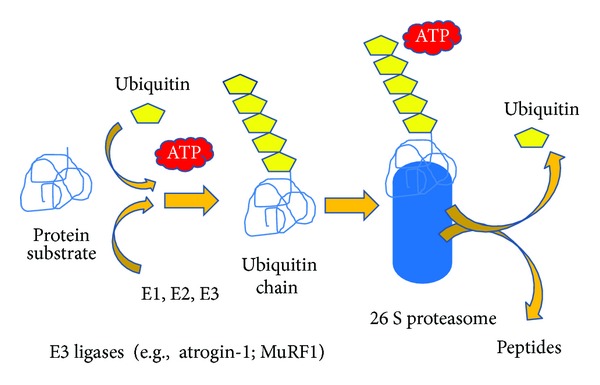
A protein designated for catabolism is bound to a series of ubiquitin (Ub) molecules in a process requiring ATP. Initially, free Ub is bound to the Ub-activating enzyme E1 in an ATP dependent process. Ub is subsequently shuttled from the Ub-activating enzyme E1 to Ub-conjugating enzyme E2 through the formation of a thioester bond between Ub and a cysteine residue of the E2 enzyme. The Ub monomer is then conjugated to the target protein through a peptide bond between the *ε*-amino group of a lysine residue in the target protein and the carboxy-terminal glycine residue in Ub via the action of a Ub-ligase enzyme E3. At least 4 Ub monomers must be attached to the protein before the target protein can be recognized and degraded by the 26S proteasome. In the degradation process, peptides are formed and the ubiquitin is released where it can be recycled again.

 In eukaryotic cells, at least five proteolytic systems are responsible for protein degradation in cells. These include autophagy, the cysteine-dependent aspartate specific proteases known as caspases [60], cathepsins [61], calcium dependent calpains [62], and the ubiquitin proteasome system (UPS) [63]. Findings from cell line, animal and human based research consistently suggest that the UPS plays a pivotal role in muscle protein catabolism. The UPS is an ATP-dependent proteolytic system that involves the degradation of specific proteins that are targeted for degradation by the addition of ubiquitin (Ub) molecules. This process is accomplished through the coordinated activity of three enzymes. Initially, free Ub is bound to the Ub-activating enzyme E1 in an ATP-dependent process. Ubiquitin is subsequently shuttled from the Ub-activating enzyme to the Ub-conjugating enzyme with the formation of a thioester bond between Ub and a cysteine residue of the E2 enzyme. Subsequently, the Ub monomer is conjugated to the target protein through a peptide bond between the *ε*-amino group of a lysine residue in the target protein and the carboxy-terminal glycine residue in Ub via the action of a Ub-ligase enzyme. For proteins degraded by the proteasome, this process is repeated until at least four Ub monomers are covalently attached via lysine residue 48 of Ub to the target protein. Once these Ub monomers are attached to the protein, the target protein can be recognized and degraded by the 26S proteasome for degradation (see Figure 3).

 Only one Ub-activating enzyme, which has been identified in sufficiently high abundance in eukaryotic cells, handles the divergent demands placed on it by the UBS. Although there are several dozen known Ub-conjugating enzymes, there are hundreds of Ub-ligases which are responsible for target specificity [64]. 

 Once the target protein is successfully ubiquitinated and recognized by the 26S proteasome, it is unfolded and fed into the proteasome in an ATP-dependent process. The 26S proteasome consists of a 20S catalytic unit and a 19S regulatory cap. Within the barrel-shaped 20 S catalytic unit, multiple alpha subunits provide structural support while multiple beta subunits exhibit chymotrypsin-like, trypsin-like or caspase-like activities that coordinately digest the protein into short oligonucleotides [65]. Exopeptidases complete the degradation of the original protein to amino acids. 

 Under conditions of atrophy, two muscle-specific Ub-ligases, atrogin-1 (known also as muscle atrophy F-box protein or MAFbx) and muscle ring finger-1 (MuRF1), as well as ubiquitin and a select group of proteasome subunits are upregulated. These conditions include burn injury [66], uremia [67], diabetes [67], denervation [68], sepsis [69], and dexamethasone administration [70].

 As noted previously, targets of Akt are the FOXO family of transcription factors that participate in the regulation of a variety of metabolic processes including protein degradation in skeletal muscle. Phosphorylation of FOXO1 and FOXO3 by Akt prevents them from translocating to the nucleus and keeps them inactive. When activated (i.e., dephosphorylated), nuclear FOXOs directly increase the expression of a variety of genes including atrogin-1 and MuRF1 E3 ligases that have been shown to be tightly linked to the muscle atrophy process. Transgenic mice that over express FOXO1 have an atrophic phenotype characterized by loss of skeletal muscle mass, impaired glycemic control, and downregulation of type I muscle fibers [71]. Mice lacking either MAFbx/atrogin 1 or MuRF1 were resistant to the effects of denervation-induced muscle atrophy compared to littermate controls [68]. This underscores the important role that FOXO1/3, MAFbx/atrogin-1, and MuRF1 play in the UPS-mediated muscle protein degradation.

 Despite the major role that the UBS plays in muscle atrophy, the sequence of events may be more complicated because the UBS alone does not appear to break down the complexes of proteins contained in actomyosin or myofibrils [72, 73]. This was suggested by Solomon and Goldberg [74] who incubated actomyosin with reconstituted components of the UBS. Under those in vitro conditions, the UBS degraded monomeric actin or myosin but not actinomycin complexes. They concluded that the disassociation of actomyocin complexes is the rate limiting step in muscle protein breakdown and that additional proteases must be present to release the constituent proteins of actinomysin. Apoptotic proteases have been identified that can cleave actin in vitro so it is logical to assume that they could be involved in muscle protein breakdown [75, 76]. Du and colleagues [77] examined caspase-3 as a possible candidate for this initial step. They incubated muscle cell and tissue lysates of actomyosin in the presence of caspase-3 and found a characteristic 14-kDa actin fragment was generated. Moreover, the products of caspase-3 action were then rapidly degraded by the UBS. Inhibiting caspase-3 activity not only blocked the breakdown of actomyosin complexes but attenuated protein degradation as well. Although other systems such as the calpains were thought to be involved in accelerating muscle proteolysis, inhibiting calcium-dependent proteases had little effect on protein degradation of myofibrillar protein in rats with chronic uremia. Thus, insulin resistance, which is present in many catabolic conditions such as CKD, activates apoptotic pathways that lead to muscle protein breakdown as a consequence of impaired PI3-K activity. 

 More recently, Cohen and colleagues [78] studied skeletal muscle from mice undergoing disuse atrophy following denervation to determine the sequence by which the MuRF1 E3 ligase induces muscle atrophy. They studied the E3 ligase with and without its RING-finger motif. The latter domain is essential for ubiquitin conjugation so that an E3 ligase lacking this motif can bind to a protein designated for degradation, but the protein cannot be ubiquitinylated. The authors uncovered that expression of the RING-fingered MuRF1 is markedly induced under conditions of denervation atrophy. In mice either lacking MuRF1 or expressing the modified MuRF1, muscle wasting is attenuated. Moreover, muscle protein breakdown occurs in an orderly sequence with loss of myosin-binding protein C, myosin light chains 1 and 2 from the myofibril before any measurable decrease in myosin heavy chain occurs. The authors concluded that thick filament disassembly was solely dependent on the ubiquitin-proteasome pathway whereas degradation of the thin filament components did not require MuRF1. Although skeletal muscle loss from disuse atrophy differs from the muscle loss associated with chronic uremia, this study does suggest that in either case that muscle protein degradation occurs in an orderly fashion and underscores the importance of the E3 ligase in promoting protein degradation. 

## 9. Angiotensin II and Insulin Resistance 

 Another factor associated with insulin resistance in chronic kidney disease is angiotensin II (Ang II). It is well known that there is activation of the renal renin-angiotensin system in CKD and this activation has deleterious effects on the heart, kidney, and vasculature. Infusion of Ang II in rats promotes loss of body weight. There is a reduction in food intake and a decrease in skeletal muscle weight. Circulating IGF-1 is markedly reduced [79, 80]. Rates of protein degradation are increased and there is activation of FOXO transcription factors, caspase 3, and the UPS. Interestingly, these changes are independent of any presser effect as mature skeletal muscle expresses little or no Ang II receptors and argues that Ang II affects muscle protein degradation indirectly through inflammatory cytokines like interleukin 6 (IL-6) [81], tumor necrosis factor-*α* (TNF-*α*) [82], serum amyloid A [81], glucocorticoids [83], and reactive oxygen species [84]. Nevertheless, when Ang II is infused in mice, 12% of total body mass and 26% of gastrocnemius muscle mass is lost in as little as 4 days [85]. These effects can be successfully reversed in muscle by the administration of 5-aminoimidazole-4-carboxamide ribonucleoside (AICAR) which reliably activates AMP-activated protein kinase (AMPK).

## 10. AMP-Activated Protein Kinase

 AMPK is a serine-threonine kinase that plays a key role in the regulation of lipid metabolism in response to metabolic stress and energy demand [86]. It is activated by AMP and it is regulated by phosphorylation. For many years, it was regarded with curiosity as it appeared to be activated under conditions of stress, but more recently it has been shown to be responsible for the phosphorylation of numerous proteins involved with cellular functions that respond to exercise and the actions of type-2 diabetes drugs [87]. As a result, a lot of interest has been generated because of the possibility that AMPK might mediate many of the health benefits of exercise in mitigating conditions related to insulin resistance including sedentary lifestyles, obesity, and aging [86]. AMPK maintains intracellular energy balance by sensing an increase in the ratio of AMP : ATP and coordinates cellular metabolic activity to provide energy in response to demand. 

 AMPK is a *αβγ* heterotrimer consisting of 7 subunits: *α*1, *α*2, *β*1, *β*2, *γ*1, *γ*2, and *γ*3 [88]. The *α* subunit contains the kinase catalytic core and is associated with the *β* subunit that functions as an anchoring subunit for *α* and *γ* while the *γ* subunit allows for AMP or ATP binding. When AMPK is activated, skeletal muscle fatty acid oxidation is increased through the phosphorylation of acetyl-CoA carboxylase and the subsequent reduction of malonyl-CoA and increase flux of long-chain fatty acyl CoA into the mitochondria [89]. In obesity, skeletal muscle fatty acid oxidation is suppressed [90] while skeletal muscle AMPK activity is reduced [91].

**Figure 4:**
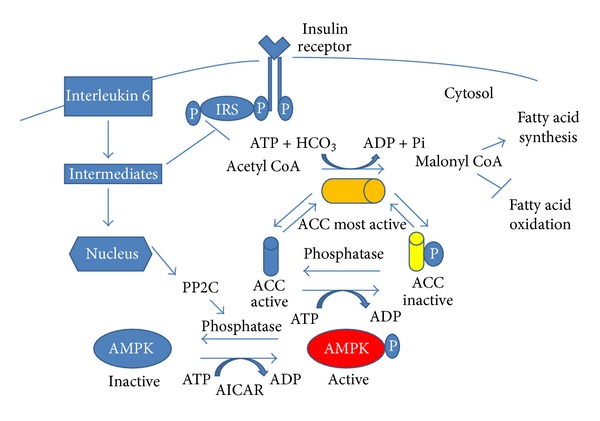
Infusion of angiotensin II (Ang II) is associated with an upregulation of various cytokines including interleukin 6 (IL-6), tumor necrosis factor (TNF), and reactive oxygen species. Chronic exposure to IL-6 impairs insulin signaling at the level of insulin receptor substrate (IRS-1) through mechanisms that involve activation of proinflammatory kinases. There is also upregulation of protein phosphatase 2Ca (PP2Ca) which is known to dephosphorylate and inactivate AMP-activated protein kinase (AMPK). Downstream targets of AMPK signaling including peroxisome-proliferator-activated receptor gamma coactivator-1*α* (PGC1-*α*) and acetyl-coenzyme A carboxylase (ACC) are reduced. When ACC is phosphorylated, it is inactive and no longer able to catalyze the synthesis of malonyl-coenzyme A. Fatty acid oxidation is blocked and muscle ATP depletion occurs.

 Protein phosphatase 2C *α* (PP2C *α*), a serine/threonine protein phosphatase, is known to dephosphorylate and inactivate AMPK. Tabony and colleagues [85] found that the inhibitory effects of Ang II on AMPK activity were mediated by the upregulation of PP2C*α*. Moreover, downstream targets of AMPK signaling, including proliferator-activated receptor-*γ* coactivator-1*α* (PGC-1*α*) and acetyl-coenzyme A carboxylase were reduced by Ang II. AICAR completely reversed the inhibitory effect of PGC-1*α* expression. As expected, acetyl-CoA carboxylase phosphorylation was decreased by Ang II, principally through a reduction in the enzyme protein, but this was not reversed by AICAR. While having no effect on acetyl-CoA carboxylase activity, AICAR did restore total acetyl-CoA carboxylase to basal levels. Thus, Ang II causes marked ATP depletion in skeletal muscle and blocks AMPK activation through a mechanism that involves increased expression of the protein phosphatase PP2C*α*. By blocking AMPK activation, Ang II blocks activation of PGC-1*α* and reduces acetyl-CoA phosphorylation. Fatty acid synthesis is turned off. When acetyl-CoA is phosphorylated, it is inactive and no longer able to catalyze the synthesis of malonyl-coenzyme A, which blocks carnitine palmitoyltransferase 1. When uninhibited, carnitine palmitoyltransferase 1 facilitates mobilization of fatty acids to the mitochondria where they can be *β*-oxidized for acute ATP production in times of metabolic stress [92, 93] (see Figure 4).

## 11. Muscle Expression of Cytokines

 As an endocrine organ, skeletal muscle expresses cytokines, also known as myokines that affect energy metabolism. These include (TNF-*α*), a pro-inflammatory cytokine that is secreted by macrophages, adipocytes and skeletal muscle [94], and interleukin-6 (IL-6) which is produced by adipocytes, immune cells, and contracting muscle [95]. IL-6 appears to play a dual role in skeletal muscle by mediating impaired insulin action in obesity and facilitating increased fuel metabolism during exercise. Elevated IL-6 levels have been reported in both adipose tissue and obesity in states of obesity and insulin resistance [96, 97]. Simultaneously, expression of IL-6 and its receptor increase with exercise. As a result, glucose uptake and utilization are enhanced by increasing GLUT4 translocation to the plasma membrane through the activation of serine/threonine protein kinase 11 (LKB1)/AMP-activated protein kinase/protein kinase B substrate of 160 kDa (AS160) pathway [96]. Note that Akt (protein kinase B) also phosphorylates AS 160 which results in GLUT4 translocation to the plasma membrane. Thus insulin and IL-6 can work synergistically acutely in enhancing glucose uptake into the cell. However, chronic exposure to IL-6 impairs insulin signaling at the level of IRS-1 by three mechanisms that involve activation of proinflammatory kinases, accumulation of suppressor of cytokine signaling 3 (SOCS3), and an increase in protein-tyrosine phosphatase 1B activity (PTP1B). The former involves phosphorylation of IRS-1 at Serine 307, in a JNK-dependent manner which is similar to that described in other insulin resistant states such as hyperinsulinemia [98] and TNF-*α* treatment [99]. When unregulated, SOCS3 can bind to the insulin receptor on a key residue for the recognition of IRS-1 and inhibit tyrosine phosphorylation [100, 101]. PTP1B dephosphorylates tyrosine residues on the insulin receptor and prevents its activation. TNF-*α* works similarly [102, 103]. TNF-*α* receptors 1 and 2 (TNFR1 and TNFR2) are located in most tissues and are upregulated with obesity [104]. 

 Plasma TNF-*α* levels generally do not change with a single bout of exercise [105]. Weight reduction through exercise training and dietary restrictions decreases plasma TNF-*α* [105]. Dietary restriction alone can decrease TNF-*α* along with other inflammatory markers. This suggests that a decrease in body fat stores plays an important role in reducing inflammation [106]. Plasma TNF-*α* levels are increased with a high fat diet and can be decreased by switching to a low-fat/high-carbohydrate diet [106]. Plasma IL-6 levels are closely linked to activity. In individuals with type 2 diabetes, increases in plasma levels of IL-6 are larger than in non-diabetic individuals [105]. With exercise training, plasma IL-6 levels decrease after exercise and muscle IL-6 receptor content is increased with exercise training. Like TNF-*α*, IL-6 plasma levels change significantly with dietary manipulation. A high fat diet leads to weight gain and increased IL-6 expression and inflammation which can be reversed by a low-fat/high-carbohydrate diet [106]. For patients who have chronic kidney disease, anemia is very common. Depending on the severity, it may preclude them from participating in a regular exercise program. Nevertheless, Kopple [107] and colleagues studied hemodialysis patients during 18 weeks of resistance and endurance training and found an increase in IGF-1 protein as well as a reduction in the 14-kDa actin fragment associated with UPS muscle protein degradation. As expected, IL-6 plasma levels dropped with endurance or resistance exercises while TNF-*α* levels remained constant. These data suggested that accelerated muscle proteolysis commonly seen in these patients could be suppressed with exercise.

 The type of exercise may be important in blunting the CKD-induced abnormalities in IGF-1 signaling. Chen et al. and Sun et al. [108, 109] studied resistance exercise (muscle overloading) in rats with CKD and found that IGF-1 as well as the downstream mediators of the IGF-1 signaling pathway, IRS-1/PI3K/p-Akt, were increased. Wang and colleagues [110] evaluated whether different types of exercise could counteract CKD-induced muscle wasting. They studied CKD mice undergoing two models of exercise, muscle overloading and treadmill running, and found that the responses were different depending upon the type of exercise. Using the plantaris muscle, the authors found that in normal mice that muscle weights increased significantly with muscle overloading or treadmill running as compared to unexercised mice. In contrast, CKD mice, plantaris muscle weights were 67% greater than in unexercised CKD mice whereas plantaris muscle weights of CKD mice undergoing treadmill running did not differ from pair-fed, unexercised CKD mice. Both types of exercise blunted CKD-induced acceleration of protein degradation. There was increased phosphorylation of Akt and FOXO1 and suppressed activation of caspase-3 and the ubiquitin-proteasome system, where the two types of exercise differed was in the degree of muscle synthesis. In mice with overloading, the decrease in muscle protein synthesis was reversed whereas there was only a slight improvement in protein synthesis in mice undergoing treadmill running which was largely attributed to a decrease in protein degradation. Thus, muscle overloading can blunt the development of muscle atrophy by suppressing protein degradation, stimulating protein synthesis, and activating progenitor cells. All of these functions can be linked to increased phosphorylation of Akt [111].

Besides loss of muscle protein through the UBS system, impaired activation and proliferation of muscle progenitor or satellite cells can result in loss of muscle protein. These cells participate in the repair of muscle in response to injury and maintain muscle protein stores. In CKD, satellite cell function is impaired in response to hormonal and metabolic changes that promote muscle wasting [112]. Whether or not these changes can be blocked by a single treatment strategy has been under intense investigation. Recently, there have been reports that inhibition of myostatin signaling interferes with muscle protein losses and improves intracellular insulin/IGF-1 signaling. 

**Figure 5:**
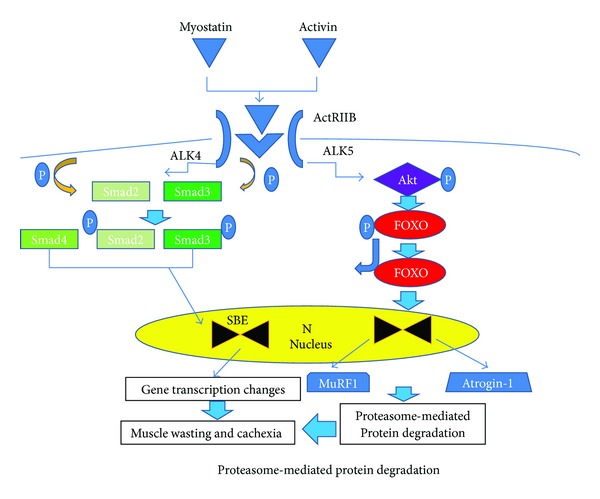
The precursor of myostatin is promyostatin and consists of a propeptide that binds to myostatin noncovalently to form an inactive complex. When myostatin is activated, it binds to its receptor; activin A bound to the extra cellular domain of a type II receptor (ActRIIB), which is present on muscle membranes. In turn ALK4 or ALK5 phosphorylates intracellular proteins called Smad2/3 and the Smad complex translocates into the nucleus and causes changes in gene transcription that ultimately result in muscle wasting and cachexia. Akt activity is also inhibited. FOXO becomes dephosphorylated and migrates to the nucleus where ubiquitin ligases MuRF1 and Atrogen 1 are synthesized. As a result, muscle proteins are degraded through the ubiquitin proteasome pathway.

Myostatin is predominately expressed in skeletal muscle and is a member of the TGF-*β* family of secreted proteins. The precursor of myostatin is promyostatin and consists of a propeptide that binds noncovalently to myostatin to form an inactive complex. Through proteolysis, action of free radicals or a decrease in pH, myostatin is activated and binds to its receptor, activin bound to the extracellular domain of a type II receptor (ActRIIB), which is present on muscle membranes [113]. In turn, activin receptor serine kinases, ALK4 or ALK5 phosphorylates intracellular proteins called Smads 2/3 and changes in gene transcription occur resulting in muscle wasting and cachexia (see Figure 5).

 The role that myostatin plays in regulating skeletal muscle mass and function is clear from deletion or knockout studies of the myostatin gene in mice which result with phenotypes with a dramatic increase in the size and number of muscle fibers [114]. A human with loss-of-function mutation who had enormous muscles has also been reported [115]. Thus, myostatin deficiency results in muscle hypertrophy and improved physical performance. 

 In disease states, myostatin protein and the activity of the myostatin/activin signaling pathway are upregulated and results in muscle wasting. Myostatin is increased in renal failure [116] and other cachexia-related disease states [117, 118]. Serum levels of Activin A, which also binds to the myostatin receptor, rise in the setting of renal failure. Both myostatin and activin A negatively influence muscle size. When mice are given myostatin or activin A, a 30% decrease in muscle mass has been recorded [119, 120]. 

 Treatment strategies for inhibiting myostatin utilizing antibodies and a peptibody, a genetically engineered myostatin-neutralizing peptide fused to Fc, have been under investigation. Zhang and colleagues [121] found that muscle atrophy was prevented in a mouse model of CKD through an increase in the rate of protein synthesis and a decrease in protein degradation. In addition, circulating levels of inflammatory cytokines including IL-6 were suppressed. Moreover, Il-6 combined with acute phase protein suppresses intracellular IGF-1 signaling and results in a decrease in the level of p-Akt. The authors further showed that treatment of cultured muscle cells with either TNF-*α* or IL-6 produced more myostatin. In CKD patients, circulating levels of TNF-*α* are high and act to increase myostatin production. In turn, IL-6 production is increased which reduces p-Akt in muscle and activates the UPS and caspase-3. Muscle atrophy follows.

## 12. Summary

 In summary, insulin resistance describes a physiological condition which is characterized by reduced tissue responses to the action of insulin for any given blood concentration of the hormone. It is a common finding in chronic kidney disease and in nondiabetic patients. It is manifested by mild fasting hyperglycemia and abnormal glucose tolerance testing during an oral or intravenous glucose load. These changes are often masked by a decline in the metabolic clearance of insulin as the glomerular filtration rate drops below 15 to 20 mL/minute. Muscle tissue accounts as the primary site for insulin resistance. The major pathway by which insulin mediates its metabolic effects is through the Class I phosphatidylinositol 3-kinase (PI3K)/Akt pathway. The basis for the impaired responses to insulin is related to the reduced activation of the downstream effector Akt caused by an increase in the amount of the PI3K regulatory p85 subunit. This is brought about through increased circulatory levels of glucocorticoids. Akt plays a critical role in regulating skeletal muscle growth and metabolism by promoting muscle hypertrophy, preventing atrophy, and suppressing apoptosis. The product of PI3K, PIP_3_, can undergo dephosphorylation through the activity of a specific phosphatase known as PTEN. This enzyme's activity is important in a number of ways. Under fasting conditions, the PI3K/Akt signaling pathway is suppressed and the formation of gluconeogenic amino acids is stimulated. PTEN is decreased in fasting in order to counteract the increase in muscle protein breakdown. In chronic diabetes as well as insulin resistance, PI3K activity is decreased while PTEN activity is increased, accelerating muscle protein breakdown. 

 Akt plays a key role in muscle metabolism by targeting the Forkhead box O or FOXO family of transcription factors for phosphorylation. As a consequence, the FOXOs are deactivated and prevented from translocating to the nucleus and activating a variety of genes that suppress skeletal muscle hypertrophy. The induction of ubiquitin ligases and muscle proteolysis, particularly of type 1 oxidative muscle fibers, is also suppressed. The FOXOs may negatively regulate skeletal muscle mass and type 1 muscle fiber-related genes through interaction and inhibition of the PGC-1*α*. PGC-1*α* plays a key role in modulating the mitochondrial network and one factor that regulates muscle fiber type determination. This is important in catabolic states such as CKD and poorly controlled diabetes. Other signaling pathways that regulate the shift in skeletal muscle fiber type have also been identified and include the calcineurin, calcium-dependent protein phosphatase. Through dephosphorylation NFAT and MEF2, transcription of prototypical muscle fiber genes that include PGC-1*α* is enhanced.

 Pathways identified with muscle protein degradation in chronic kidney disease include activation of the ubiquitin-proteasome system. Proteins identified through a group of enzymes called E3 ubiquitin ligases (Atrogin 1 and MuRF 1) for protein degradation are bound by ubiquitin and taken to the proteasome where they are degraded. As noted, the targets of Akt are the FOXO family of transcription factors. When phosphorylated they are inactive. When dephosphorylated, the FOXOs directly increase the expression of a variety of genes including atrogin 1 and MurF 1 which have been shown to be tightly linked to the muscle atrophy process. 

 Another factor associated with insulin resistance in chronic kidney disease is Ang II where there is activation of the renal renin-angiotensin system. Infusion of Ang II causes an increase in rates of muscle protein degradation, activation of FOXO transcription factors and the UPS. As there are no Ang II receptors in muscle, it is thought that the effects of Ang II in muscle are indirect through inflammatory cytokines or reactive oxygen species. In the setting of Ang II, (PP2C *α*) is upregulated and dephosphorylates and inactivates AMPK as skeletal muscle ATP is depleted. Downstream targets of AMPK signaling such as PGC-1*α* and acetyl-coenzyme A carboxylase are also reduced. AICAR completely reverses the inhibitory effect on PGC-1*α* and restores total acetyl-CoA carboxylase to basal levels. 

 Skeletal muscle expresses cytokines that affect energy metabolism and include TNF-*α* and Il-6. The latter plays a dual role in skeletal muscle. It mediates impaired insulin action in obesity while facilitating increased glucose uptake and its utilization during exercise through a mechanism involving GLUT4. Interestingly, chronic exposure to interleukin-6 impairs insulin signaling at the level of IRS-1. TNF-*α* works similarly and its receptors, which are located in most tissues, are upregulated with obesity. Weight reduction reduces levels of both TNF-*α* and IL-6 while exercise reduces IL-6. For patients with CKD, the type of exercise may be important in blocking abnormalities in IGF-1 signaling. This has important ramifications whereby buildup of muscle may positively impact functional status. 

 Myostatin and activin A, which binds to the myostatin receptor, have been linked to muscle atrophy and are upregulated in renal failure. Treatment strategies for inhibiting myostatin utilizing antibodies and a peptibody have shown promise in preventing muscle atrophy in a mouse model of CKD. 

## Abbreviations

ACC: Activated acetyl-coenzyme A carboxylase
ActRIIB: Activin A bound to the extra cellular domain of a type II receptor
AICAR: 5-Aminoimidazole-4-carboxamide ribonucleoside AICAR
Akt: Protein kinase B
ALK 4: Activin receptor-like kinase 4 or Activin A Receptor, type 1B
AMPK: AMP-activated protein kinase
Ang II: Angiotensin II
atrogin-1: Muscle atrophy F-box protein or MAFbx
CKD: Chronic kidney disease
GLUT4: Glucose transporter type 4
IGF: Insulin growth factor
IL-6: Interleukin-6
IR: Insulin receptor
IRS: Insulin receptor substrate
MCIP1.4: Modulatory calcineurin interacting protein 1.4
MEF2: Myocytes enhancer factor 2
MEK/ERK: Mitogen activated protein kinase pathway/extracellular signal related kinase pathway.
MRF4: Myogenic regulatory factor 4
MuRF1: Muscle ring finger-1
NFAT: Nuclear factor of activated T cells
PGC1-*α*: Transcriptional coactivator peroxisome-proliferator-activated receptor gamma coactivator-1*α*
PIP2: Phosphatidylinositol (3, 4)-biphosphate
PIP3: Phosphatidylinositol (3, 4, 5)-triphosphate
PI3K: Class I phosphatidylinositol 3-Kinase
PP2C*α*: Protein phosphatase 2C*α*
PTEN: Phosphatase and tensin homolog deleted from chromosome 10
PTP1B: Protein-tyrosine phosphatase 1B activity
SOCS3: Suppressor of cytokine signaling 3
TNF-*α*: Tumor necrosis factor-*α*
TNFR1 and TNFR2: TNF-*α* receptors 1 and 2
Ub: Ubiquitin
UPS: Ubiquitin proteasome system.

## References

[1] DeFronzo RA, Alvestrand A, Smith D, Hendler R, Hendler E, Wahren J (1981). Insulin resistance in uremia. *Journal of Clinical Investigation*.

[2] Mak RHK, Haycock GB, Chantler C (1983). Glucose intolerance in children with chronic renal failure. *Kidney International*.

[3] Rabkin R, Simon NM, Steiner S, Colwell JA (1970). Effect of renal disease on renal uptake and excretion of insulin in man. *New England Journal of Medicine*.

[4] Mondon CE, Dolkas CB, Reaven GM (1978). Effect of acute uremia on insulin removal by the isolated perfused rat liver and muscle. *Metabolism: Clinical and Experimental*.

[5] Cecchin F, Ittoop O, Sinha MK, Caro JF (1988). Insulin resistance in uremia: insulin receptor kinase activity in liver and muscle from chronic uremic rats. *American Journal of Physiology*.

[6] Tsao T, Fervenza F, Friedlaender M, Chen Y, Rabkin R (2002). Effect of prolonged uremia on insulin-like growth factor-I receptor autophosphorylation and tyrosine kinase activity in kidney and muscle. *Experimental Nephrology*.

[7] Ding H, Gao XL, Hirschberg R, Vadgama JV, Kopple JD (1996). Impaired actions of insulin-like growth factor 1 on protein synthesis and degradation in skeletal muscle of rats with chronic renal failure: evidence for a postreceptor defect. *Journal of Clinical Investigation*.

[8] Bailey JL, Zheng B, Hu Z, Price SR, Mitch WE (2006). Chronic kidney disease causes defects in signaling through the insulin receptor substrate/phosphatidylinositol 3-kinase/Akt pathway: implications for muscle atrophy. *Journal of the American Society of Nephrology*.

[9] Franch HA, Price SR (2005). Molecular signaling pathways regulating muscle proteolysis during atrophy. *Current Opinion in Clinical Nutrition and Metabolic Care*.

[10] Lee SW, Dai G, Hu Z, Wang X, Du J, Mitch WE (2004). Regulation of muscle protein degradation: coordinated control of apoptotic and ubiquitin-proteasome systems by phosphatidylinositol 3 kinase. *Journal of the American Society of Nephrology*.

[11] Sacheck JM, Ohtsuka A, McLary SC, Goldberg AL (2004). IGF-I stimulates muscle growth by suppressing protein breakdown and expression of atrophy-related ubiquitin ligases, atrogin-1 and MuRF1. *American Journal of Physiology*.

[12] Saad MJA, Folli F, Kahn JA, Kahn CR (1993). Modulation of insulin receptor, insulin receptor substrate-1, and phosphatidylinositol 3-kinase in liver and muscle of dexamethasone-treated rats. *Journal of Clinical Investigation*.

[13] Giorgino F, Pedrini MT, Matera L, Smithi RJ (1997). Specific increase in p85*α* expression in response to dexamethasone is associated with inhibition of insulin-like growth factor-I stimulated phosphatidylinositol 3-kinase activity in cultured muscle cells. *Journal of Biological Chemistry*.

[14] Giorgino F, Almahfouz A, Goodyear LJ, Smith RJ (1993). Glucocorticoid regulation of insulin receptor and substrate IRS-1 tyrosine phosphorylation in rat skeletal muscle in vivo. *Journal of Clinical Investigation*.

[15] May RC, Kelly RA, Mitch WE (1987). Mechanisms for defects in muscle protein metabolism in rats with chronic uremia. Influence of metabolic acidosis. *Journal of Clinical Investigation*.

[16] Vanhaesebroeck B, Alessi DR (2000). The PI3K-PBK1 connection: more than just a road to PKB. *Biochemical Journal*.

[17] Sable CL, Filippa N, Hemmings B, van Obberghen E (1997). cAMP stimulates protein kinase B in a Wortmannin-insensitive manner. *FEBS Letters*.

[18] Ueki K, Yamamoto-Honda R, Kaburagi Y (1998). Potential role of protein kinase B in insulin-induced glucose transport, glycogen synthesis, and protein synthesis. *Journal of Biological Chemistry*.

[19] Whiteman EL, Cho H, Birnbaum MJ (2002). Role of Akt/protein kinase B in metabolism. *Trends in Endocrinology and Metabolism*.

[20] Cho H, Thorvaldsen JL, Chu Q, Feng F, Birnbaum MJ (2001). Akt1/PKB*α* is required for normal growth but dispensable for maintenance of glucose homeostasis in mice. *Journal of Biological Chemistry*.

[21] Cho H, Mu J, Kim JK (2001). Insulin resistance and a diabetes mellitus-like syndrome in mice lacking the protein kinase Akt2 (PKB*β*). *Science*.

[22] Easton RM, Cho H, Roovers K (2005). Role for Akt3/protein kinase by in attainment of normal brain size. *Molecular and Cellular Biology*.

[23] Wan M, Easton RM, Gleason CE (2012). Loss of Akt 1 in mice increases energy expenditure and protects against diet-induced obesity. *Molecular and Cellular Biology*.

[24] Bodine SC, Stitt TN, Gonzalez M (2001). Akt/mTOR pathway is a crucial regulator of skeletal muscle hypertrophy and can prevent muscle atrophy in vivo. *Nature Cell Biology*.

[25] Chen WS, Xu PZ, Gottlob K (2001). Growth retardation and increased apoptosis in mice with homozygous disruption of the akt1 gene. *Genes and Development*.

[26] Dupont J, Renou JP, Shani M, Hennighausen L, LeRoith D (2002). PTEN overexpression suppresses proliferation and differentiation and enhances apoptosis of the mouse mammary epithelium. *Journal of Clinical Investigation*.

[27] Lo YT, Tsao CJ, Liu IM, Liou SS, Cheng JT (2004). Increase of PTEN gene expression in insulin resistance. *Hormone and Metabolic Research*.

[28] Wijesekara N, Konrad D, Eweida M (2005). Muscle-specific Pten deletion protects against insulin resistance and diabetes. *Molecular and Cellular Biology*.

[29] Hu Z, In HL, Wang X (2007). PTEN expression contributes to the regulation of muscle protein degradation in diabetes. *Diabetes*.

[30] Accili D, Arden KC (2004). FoxOs at the crossroads of cellular metabolism, differentiation, and transformation. *Cell*.

[31] Ayala JE, Streeper RS, Desgrosellier JS (1999). Conservation of an insulin response unit between mouse and human glucose-6-phosphatase catalytic subunit gene promoters: transcription factor FKHR binds the insulin response sequence. *Diabetes*.

[32] Nakae J, Kitamura T, Silver DL, Accili D (2001). The forkhead transcription factor Foxo1 (Fkhr) confers insulin sensitivity onto glucose-6-phosphatase expression. *Journal of Clinical Investigation*.

[33] Medema RH, Kops GJPL, Bos JL, Burgering BMT (2000). AFX-like Forkhead transcription factors mediate cell-cycle regulation by Ras and PKB through p27(kip1). *Nature*.

[34] Kops GJPL, Dansen TB, Polderman PE (2002). Forkhead transcription factor FOXO3a protects quiescent cells from oxidative stress. *Nature*.

[35] Chakravarthy MV, Davis BS, Booth FW (2000). IGF-I restores satellite cell proliferative potential in immobilized old skeletal muscle. *Journal of Applied Physiology*.

[36] Sandri M, Sandri C, Gilbert A (2004). Foxo transcription factors induce the atrophy-related ubiquitin ligase atrogin-1 and cause skeletal muscle atrophy. *Cell*.

[37] Stitt TN, Drujan D, Clarke BA (2004). The IGF-1/PI3K/Akt pathway prevents expression of muscle atrophy-induced ubiquitin ligases by inhibiting FOXO transcription factors. *Molecular Cell*.

[38] Kamei Y, Miura S, Suzuki M (2004). Skeletal muscle FOXO1 (FKHR) transgenic mice have less skeletal muscle mass, down-regulated type I (slow twitch/red muscle) fiber genes, and impaired glycemic control. *Journal of Biological Chemistry*.

[39] Lin J, Wu H, Tarr PT (2002). Transcriptional co-activator PGC-1*α* drives the formation of slow-twitch muscle fibres. *Nature*.

[40] Zechner C, Lai L, Zechner JF (2010). Total skeletal muscle PGC-1 deficiency uncouples mitochondrial derangements from fiber type determination and insulin sensitivity. *Cell Metabolism*.

[41] Lowell BB, Shulman GI (2005). Mitochondrial dysfunction and type 2 diabetes. *Science*.

[42] Morino K, Petersen KF, Dufour S (2005). Reduced mitochondrial density and increased IRS-1 serine phosphorylation in muscle of insulin-resistant offspring of type 2 diabetic parents. *Journal of Clinical Investigation*.

[43] Petersen KF, Dufour S, Morino K, Yoo PS, Cline GW, Shulman GI (2012). Reversal of muscle Insulin resistance by weight reduction in young, lean, insulin-resistant offspring of parents with type 2 diabetes. *Proceedings of the National Academy of Sciences of the United States of America*.

[44] Schiaffino S (2010). Fibre types in skeletal muscle: a personal account. *Acta Physiologica*.

[45] Naya FJ, Mercer B, Shelton J, Richardson JA, Williams RS, Olson EN (2000). Stimulation of slow skeletal muscle fiber gene expression by calcineurin in vivo. *Journal of Biological Chemistry*.

[46] Wu H, Kanatous SB, Thurmond FA (2002). Regulation of mitochondrial biogenesis in skeletal muscle by caMK. *Science*.

[47] Chin ER, Olson EN, Richardson JA (1998). A calcineurin-dependent transcriptional pathway controls skeletal muscle fiber type. *Genes and Development*.

[48] Hogan PG, Chen L, Nardone J, Rao A (2003). Transcriptional regulation by calcium, calcineurin, and NFAT. *Genes and Development*.

[49] Roberts-Wilson TK, Reddy RN, Bailey JL (2010). Calcineurin signaling and PGC-1*α* expression are suppressed during muscle atrophy due to diabetes. *Biochimica et Biophysica Acta*.

[50] Bailey JL, Wang X, England BK, Price SR, Ding X, Mitch WE (1996). The acidosis of chronic renal failure activates muscle proteolysis in rats by augmenting transcription of genes encoding proteins of the ATP-dependent ubiquitin-proteasome pathway. *Journal of Clinical Investigation*.

[51] Price SR, Bailey JL, Wang X (1996). Muscle wasting in insulinopenic rats results from activation of the ATP-dependent, ubiquitin-proteasome proteolytic pathway by a mechanism including gene transcription. *Journal of Clinical Investigation*.

[52] Song YH, Li Y, Du J, Mitch WE, Rosenthal N, Delafontaine P (2005). Muscle-specific expression of IGF-1 blocks angiotensin II-induced skeletal muscle wasting. *Journal of Clinical Investigation*.

[53] Stenvinkel P, Heimbürger O, Paultre F (1999). Strong association between malnutrition, inflammation, and atherosclerosis in chronic renal failure. *Kidney International*.

[54] May RC, Bailey JL, Mitch WE, Masud T, England BK (1996). Glucocorticoids and acidosis stimulate protein and amino acid catabolism in vivo. *Kidney International*.

[55] May RC, Kelly RA, Mitch WE (1986). Metabolic acidosis stimulates protein degradation in rat muscle by a glucocorticoid-dependent mechanism. *Journal of Clinical Investigation*.

[56] Mak RHK (1996). Insulin resistance but IGF-I sensitivity in chronic renal failure. *American Journal of Physiology*.

[57] Reaich D, Graham KA, Channon SM (1995). Insulin-mediated changes in PD and glucose uptake after correction of acidosis in humans with CRF. *American Journal of Physiology*.

[58] Mak RHK (1998). Effect of metabolic acidosis on insulin action and secretion in uremia. *Kidney International*.

[59] Garibotto G, Russo R, Sofia A (1994). Skeletal muscle protein synthesis and degradation in patients with chronic renal failure. *Kidney International*.

[60] Lecker SH, Mitch WE (2011). Proteolysis by the ubiquitin-proteasome system and kidney disease. *Journal of the American Society of Nephrology*.

[61] Larbaud D, Balage M, Taillandier D, Combaret L, Grizard J, Attaix D (2001). Differential regulation of the lysosomal, Ca^2+^-dependent and ubiquitin/proteasome-dependent proteolytic pathways in fast-twitch and slow-twitch rat muscle following hyperinsulinaemia. *Clinical Science*.

[62] Bartoli M, Richard I (2005). Calpains in muscle wasting. *International Journal of Biochemistry and Cell Biology*.

[63] Murton AJ, Constantin D, Greenhaff PL (2008). The involvement of the ubiquitin proteasome system in human skeletal muscle remodelling and atrophy. *Biochimica et Biophysica Acta*.

[64] Patton EE, Willems AR, Tyers M (1998). Combinatorial control in ubiquitin-dependent proteolysis: don’t Skp the F-box hypothesis. *Trends in Genetics*.

[65] Attaix D, Ventadour S, Codran A, Béchet D, Taillandier D, Combaret L (2005). The ubiquitin-proteasome system and skeletal muscle wasting. *Essays in Biochemistry*.

[66] Lang CH, Huber D, Frost RA (2007). Burn-induced increase in atrogin-1 and MuRF-1 in skeletal muscle is glucocorticoid independent but downregulated by IGF-I. *American Journal of Physiology*.

[67] Lecker SH, Jagoe RT, Gilbert A (2004). Multiple types of skeletal muscle atrophy involve a common program of changes in gene expression. *FASEB Journal*.

[68] Bodine SC, Latres E, Baumhueter S (2001). Identification of ubiquitin ligases required for skeletal Muscle Atrophy. *Science*.

[69] Li YP, Chen Y, John J (2005). TNF-*α* acts via p38 MAPK to stimulate expression of the ubiquitin ligase atrogin1/MAFbx in skeletal muscle. *FASEB Journal*.

[70] Clarke BA, Drujan D, Willis MS (2007). The E3 ligase MuRF1 degrades myosin heavy chain protein in dexamethasone-treated skeletal muscle. *Cell Metabolism*.

[71] Kamei Y, Miura S, Suzuki M (2004). Skeletal muscle FOXO1 (FKHR) transgenic mice have less skeletal muscle mass, down-regulated type I (slow twitch/red muscle) fiber genes, and impaired glycemic control. *Journal of Biological Chemistry*.

[72] Mitch WE, Goldberg AL (1996). Mechanisms of disease: mechanisms of muscle wasting: the role of the ubiquitin-proteasome pathway. *New England Journal of Medicine*.

[73] Lecker SH, Solomon V, Mitch WE, Goldberg AL (1999). Muscle protein breakdown and the critical role of the ubiquitin-proteasome pathway in normal and disease states. *Journal of Nutrition*.

[74] Solomon V, Goldberg AL (1996). Importance of the ATP-ubiquitin-proteasome pathway in the degradation of soluble and myofibrillar proteins in rabbit muscle extracts. *Journal of Biological Chemistry*.

[75] Mashima T, Naito M, Noguchi K, Miller DK, Nicholson DW, Tsuruo T (1997). Actin cleavage by CPP-32/apopain during the development of apoptosis. *Oncogene*.

[76] Kayalar C, Örd T, Testa MP, Zhong LT, Bredesen DE (1996). Cleavage of actin by interleukin 1*β*-converting enzyme to reverse DNase I inhibition. *Proceedings of the National Academy of Sciences of the United States of America*.

[77] Du J, Wang X, Miereles C (2004). Activation of caspase-3 is an initial step triggering accelerated muscle proteolysis in catabolic conditions. *Journal of Clinical Investigation*.

[78] Cohen S, Brault JJ, Gygi SP (2009). During muscle atrophy, thick, but not thin, filament components are degraded by MuRF1-dependent ubiquitylation. *Journal of Cell Biology*.

[79] Brink M, Price SR, Chrast J (2001). Angiotensin II induces skeletal muscle wasting through enhanced protein degradation and down-regulates autocrine insulin-like growth factor I. *Endocrinology*.

[80] Song YH, Li Y, Du J, Mitch WE, Rosenthal N, Delafontaine P (2005). Muscle-specific expression of IGF-1 blocks angiotensin II-induced skeletal muscle wasting. *Journal of Clinical Investigation*.

[81] Zhang L, Du J, Hu Z (2009). IL-6 and serum amyloid a synergy mediates angiotensin II-induced muscle wasting. *Journal of the American Society of Nephrology*.

[82] Zera T, Ufnal M, Szczepanska-Sadowska E (2008). Central TNF-*α* elevates blood pressure and sensitizes to central pressor action of angiotensin II in the infarcted rats. *Journal of Physiology and Pharmacology*.

[83] Brink M, Anwar A, Delafontaine P (2002). Neurohormonal factors in the development of catabolic/anabolic imbalance and cachexia. *International Journal of Cardiology*.

[84] Mitsuishi M, Miyashita K, Muraki A, Itoh H (2009). Angiotensin II reduces mitochondrial content in skeletal muscle and affects glycemic control. *Diabetes*.

[85] Tabony AM, Yoshida T, Galvez S (2011). Angiotensin II upregulates protein phosphatase 2C alpha and inhibits AMP-activated protein kinase signaling and energy balance leading to skeletal muscle wasting. *Hypertension*.

[86] Steinberg GR, Macaulay SL, Febbraio MA, Kemp BE (2006). AMP-activated protein kinase—the fat controller of the energy railroad. *Canadian Journal of Physiology and Pharmacology*.

[87] Kahn BB, Alquier T, Carling D, Hardie DG (2005). AMP-activated protein kinase: ancient energy gauge provides clues to modern understanding of metabolism. *Cell Metabolism*.

[88] Kemp BE, Stapleton D, Campbell DJ (2003). AMP-activated protein kinase, super metabolic regulator. *Biochemical Society Transactions*.

[89] Ruderman NB, Saha AK, Vavvas D, Witters LA (1999). Malonyl-CoA, fuel sensing, and insulin resistance. *American Journal of Physiology*.

[90] Gaster M, Rustan AC, Aas V, Beck-Nielsen H (2004). Reduced lipid oxidation in skeletal muscle from type 2 diabetic subjects may be of genetic origin: evidence from cultured myotubes. *Diabetes*.

[91] Bandyopadhyay GK, Yu JG, Ofrecio J, Olefsky JM (2006). Increased malonyl-CoA levels in muscle from obese and type 2 diabetic subjects lead to decreased fatty acid oxidation and increased lipogenesis; thiazolidinedione treatment reverses these defects. *Diabetes*.

[92] Steinberg GR, Michell BJ, van Denderen BJW (2006). Tumor necrosis factor *α*-induced skeletal muscle insulin resistance involves suppression of AMP-kinase signaling. *Cell Metabolism*.

[93] McGee SL, Hargreaves M (2010). AMPK-mediated regulation of transcription in skeletal muscle. *Clinical Science*.

[94] Stefanyk LE, Dyck DJ (2010). The interaction between adipokines, diet and exercise on muscle insulin sensitivity. *Current Opinion in Clinical Nutrition and Metabolic Care*.

[95] Fischer CP (2006). Interleukin-6 in acute exercise and training: what is the biological relevance?. *Exercise Immunology Review*.

[96] Nieto-Vazquez I, Fernandez-Veledo S, de Alvaro C, Lorenzo M (2008). Dual role of interleukin-6 in regulating insulin sensitivity in murine skeletal muscle. *Diabetes*.

[97] Pedersen BK, Febbraio MA (2008). Muscle as an endocrine organ: focus on muscle-derived interleukin-6. *Physiological Reviews*.

[98] Rui L, Aguirre V, Kim JK (2001). Insulin/IGF-1 and TNF-*α* stimulate phosphorylation of IRS-1 at inhibitory Ser307 via distinct pathways. *Journal of Clinical Investigation*.

[99] de Alvaro C, Teruel T, Hernandez R, Lorenzo M (2004). Tumor necrosis factor *α* produces insulin resistance in skeletal muscle by activation of inhibitor *κ*B kinase in a p38 MAPK-dependent manner. *Journal of Biological Chemistry*.

[100] Rieusset J, Bouzakri K, Chevillotte E (2004). Suppressor of cytokine signaling 3 expression and insulin resistance in skeletal muscle of obese and type 2 diabetic patients. *Diabetes*.

[101] Ueki K, Kondo T, Kahn CR (2004). Suppressor of cytokine signaling 1 (SOCS-1) and SOCS-3 cause insulin resistance through inhibition of tyrosine phosphorylation of insulin receptor substrate proteins by discrete mechanisms. *Molecular and Cellular Biology*.

[102] Nieto-Vazquez I, Fernández-Veledo S, de Alvaro C, Rondinone CM, Valverde AM, Lorenzo M (2007). Protein-tyrosine phosphatase 1B-deficient myocytes show increased insulin sensitivity and protection against tumor necrosis factor-*α*-induced insulin resistance. *Diabetes*.

[103] Zabolotny JM, Kim YB, Welsh LA, Kershaw EE, Neel BG, Kahn BB (2008). Protein-tyrosine phosphatase 1B expression is induced by inflammation in vivo. *Journal of Biological Chemistry*.

[104] Zahorska-Markiewicz B, Janowska J, Olszanecka-Glinianowicz M, Zurakowski A (2000). Serum concentrations of TNF-*α* and soluble TNF-*α* receptors in obesity. *International Journal of Obesity*.

[105] Febbraio MA, Steensberg A, Starkie RL, McConell GK, Kingwell BA (2003). Skeletal muscle interleukin-6 and tumor necrosis factor-*α* release in healthy subjects and patients with type 2 diabetes at rest and during exercise. *Metabolism: Clinical and Experimental*.

[106] Lee IS, Shin G, Choue R (2010). Shifts in diet from high fat to high carbohydrate improved levels of adipokines and pro-inflammatory cytokines in mice fed a high-fat diet. *Endocrine Journal*.

[107] Kopple JD, Wang H, Casaburi R (2007). Exercise in maintenance hemodialysis patients induces transcriptional changes in genes favoring anabolic muscle. *Journal of the American Society of Nephrology*.

[108] Chen Y, Sood S, Biada J, Roth R, Rabkin R (2008). Increased workload fully activates the blunted IRS-1/PI3-kinase/Akt signaling pathway in atrophied uremic muscle. *Kidney International*.

[109] Sun DF, Chen Y, Rabkin R (2006). Work-induced changes in skeletal muscle IGF-1 and myostatin gene expression in uremia. *Kidney International*.

[110] Wang XH, Du J, Klein JD, Bailey JL, Mitch WE (2009). Exercise ameliorates chronic kidney disease-induced defects in muscle protein metabolism and progenitor cell function. *Kidney International*.

[111] Lecker SH, Goldberg AL, Mitch WE (2006). Protein degradation by the ubiquitin-proteasome pathway in normal and disease states. *Journal of the American Society of Nephrology*.

[112] Schwarzkopf M, Coletti D, Sassoon D, Marazzi G (2006). Muscle cachexia is regulated by a p53-PW1/Peg3-dependent pathway. *Genes and Development*.

[113] Lee SJ (2008). Genetic analysis of the role of proteolysis in the activation of latent myostatin. *PLoS ONE*.

[114] McPherron AC, Lawler AM, Lee SJ (1997). Regulation of skeletal muscle mass in mice by a new TGF-*β* superfamily member. *Nature*.

[115] Schuelke M, Wagner KR, Stolz LE (2004). Myostatin mutation associated with gross muscle hypertrophy in a child. *New England Journal of Medicine*.

[116] Sun DF, Chen Y, Rabkin R (2006). Work-induced changes in skeletal muscle IGF-1 and myostatin gene expression in uremia. *Kidney International*.

[117] Gonzalez-Cadavid NF, Taylor WE, Yarasheski K (1998). Organization of the human myostatin gene and expression in healthy men and HIV-infected men with muscle wasting. *Proceedings of the National Academy of Sciences of the United States of America*.

[118] Gruson D, Ahn SA, Ketelslegers JM, Rousseau MF (2011). Increased plasma myostatin in heart failure. *European Journal of Heart Failure*.

[119] Zimmers TA, Davies MV, Koniaris LG (2002). Induction of cachexia in mice by systemically administered myostatin. *Science*.

[120] Zhou X, Wang JL, Lu J (2010). Reversal of cancer cachexia and muscle wasting by ActRIIB antagonism leads to prolonged survival. *Cell*.

[121] Zhang L, Rajan V, Lin E (2011). Pharmacological inhibition of myostatin suppresses systemic inflammation and muscle atrophy in mice with chronic kidney disease. *FASEB Journal*.

